# Exploiting DNA repair pathways for tumor sensitization, mitigation of resistance, and normal tissue protection in radiotherapy

**DOI:** 10.20517/cdr.2020.89

**Published:** 2021-06-19

**Authors:** Jac A. Nickoloff, Lynn Taylor, Neelam Sharma, Takamitsu A. Kato

**Affiliations:** Department of Environmental and Radiological Health Sciences, Colorado State University, Ft. Collins, CO 80523, USA.

**Keywords:** DNA repair, DNA double-strand break repair, non-homologous end-joining, homologous recombination, radiosensitization, radioprotection, cancer therapy

## Abstract

More than half of cancer patients are treated with radiotherapy, which kills tumor cells by directly and indirectly inducing DNA damage, including cytotoxic DNA double-strand breaks (DSBs). Tumor cells respond to these threats by activating a complex signaling network termed the DNA damage response (DDR). The DDR arrests the cell cycle, upregulates DNA repair, and triggers apoptosis when damage is excessive. The DDR signaling and DNA repair pathways are fertile terrain for therapeutic intervention. This review highlights strategies to improve therapeutic gain by targeting DDR and DNA repair pathways to radiosensitize tumor cells, overcome intrinsic and acquired tumor radioresistance, and protect normal tissue. Many biological and environmental factors determine tumor and normal cell responses to ionizing radiation and genotoxic chemotherapeutics. These include cell type and cell cycle phase distribution; tissue/tumor microenvironment and oxygen levels; DNA damage load and quality; DNA repair capacity; and susceptibility to apoptosis or other active or passive cell death pathways. We provide an overview of radiobiological parameters associated with X-ray, proton, and carbon ion radiotherapy; DNA repair and DNA damage signaling pathways; and other factors that regulate tumor and normal cell responses to radiation. We then focus on recent studies exploiting DSB repair pathways to enhance radiotherapy therapeutic gain.

## INTRODUCTION

Ionizing radiation has been used to treat cancer for more than 120 years, and radiotherapy is widely used to treat many types of cancer. More than half of cancer patients receive radiation as monotherapy or in combination with surgery, genotoxic chemotherapy, and targeted therapy. Radiation is usually delivered with external beams, but radioactive implants (brachytherapy) are used to treat prostate, head and neck, breast, eye, and other cancers^[1]^. Regardless of the mode of delivery, ionizing radiation is effective because it causes cytotoxic DNA damage (i.e., it is genotoxic), and in this way it is similar to genotoxic chemotherapy. However, radiotherapy is only effective for local tumor control and isolated metastases, whereas genotoxic chemotherapy, delivered systemically, can also treat widespread metastatic disease. There is evidence that radiotherapy may be effective against distant disease, through immune-mediated, non-targeted abscopal effects, but this approach is currently limited to pre-clinical studies^[2]^. Radiotherapy has several benefits for patients: It is non-invasive, painless, and has low rates of severe side-effects, highlighting another difference from systemic, genotoxic chemotherapy which often causes side effects that compromise patient quality of life. Although metastatic disease is ultimately responsible for most cancer deaths, the importance of local tumor control should not be underestimated. As noted in a widely used radiation oncology textbook, “…for tumors with high metastatic potential, such as breast, prostate, and lung…improved locoregional control by radiotherapy with or without chemotherapy enhances overall [patient] survival”^[3]^. Among the ongoing challenges in the radiotherapy field are the adverse effects of radiation on sensitive, normal tissues adjacent to tumors, in particular brain, spinal cord, and heart. In contrast, systemic genotoxins cause widespread damage, in particular to proliferative normal tissues including gastrointestinal lining and bone marrow, causing nausea and anemia, as well as non-proliferating brain tissue, causing chemotherapy-induced cognitive impairment or “chemo-brain”^[4]^. For both genotoxic chemotherapeutics and radiation, there is great interest in understanding mechanisms of intrinsic and acquired tumor cell resistance to these agents^[5–8]^.

The goal of radiotherapy is to completely eradicate tumor cells while sparing nearby normal tissue. The efficacy of radiotherapy has greatly improved with the development of advanced techniques for diagnostic imaging, beam-focusing, and beam-shaping^[9,10]^, and treatment outcomes continue to improve as combination therapeutic strategies mature^[11]^. Two ways that combination therapies can improve therapeutic gain are to radiosensitize tumor cells, especially those with high intrinsic or acquired radioresistance, and protect normal tissue. There are many biological parameters that modulate tumor and normal cell responses to radiation, such as cell type, cell cycle phase, tissue/tumor microenvironment, oxygen levels, DNA repair capacity, and others. We begin with a synopsis of radiation damage to cellular components; cellular responses to radiation damage; environmental and cellular factors that determine normal and tumor cell radiosensitivity; and strategies used to counter tumor radioresistance or protect normal tissue from radiation damage. We then discuss how DNA repair and DNA damage response (DDR) pathways can be exploited to radiosensitize tumor cells and protect normal tissue during radiotherapy.

## IONIZING RADIATION DAMEGE TO CELLULAR COMPONENTS AND CELL RESPONSES

Genotoxic chemotherapeutics and ionizing radiation kill cells by directly or indirectly damaging DNA or interfering with DNA metabolism (DNA polymerases, topoisomerases, or chromosome segregation machinery). Ionizing radiation, whether delivered by X-rays, protons, or carbon ions, causes damage to cellular components through direct energy absorption or indirectly by ionizing water to generate reactive oxygen species (ROS), including hydroxyl radicals, superoxide, and hydrogen peroxide^[12]^. ROS are highly reactive and interact almost immediately with cellular components, causing oxidative and other damage to proteins, nucleic acids, and membrane components. ROS are also generated during normal cell metabolism, primarily from mitochondrial function^[13,14]^. Cells survive and thrive despite > 100,000 spontaneous DNA lesions/cell/day, including ~10,000 single-strand breaks and ~50 DNA double-strand breaks (DSBs)^[15–17]^.

Nearly all DNA lesions block DNA replication, although some can be bypassed by error-prone translesion DNA polymerases^[18]^. The ability of cells to manage this remarkable daily lesion load is a reflection of the high efficiency of DNA repair systems. That said, DNA damage can cause mutations, chromosome structural alterations, cell cycle arrest, senescence, and cell death. Among the hundreds of types of DNA lesions, DSBs are among the most cytotoxic, and the cytotoxicity of genotoxic chemicals and ionizing radiation is largely due to DSBs^[19,20]^. Other double-strand lesions, such as inter-strand crosslinks, are also highly cytotoxic^[21]^.

Cells respond to DNA damage by activating checkpoint signaling and DNA repair pathways, collectively termed the DDR. DDR promotes cell survival and suppresses cancer by promoting genome stability, but it also triggers programmed cell death when damage is excessive. Altered expression or mutation of DDR proteins predispose to cancer, determine tumor response to chemo- and radiotherapy, and underlie several congenital conditions including multiple types of Seckel syndrome, primordial dwarfism, and premature aging syndromes^[22–24]^. The DDR is a major determinant of cancer cell responses to chemo- and radiotherapy, and is thus an enticing target to augment cancer therapy^[25–30]^. DDR components are often defective in cancer, but because the DDR is a complex network of interacting/cross-talking pathways, cells can respond to alterations in one pathway with compensatory changes in other pathways. Compensatory pathways within the DDR network represent formidable obstacles to successful cancer treatment. A better understanding of DDR pathways can reveal synthetic lethal relationships that can be exploited to augment cancer therapy in general, and to develop personalized therapies^[31–35]^.

**Figure 1.**
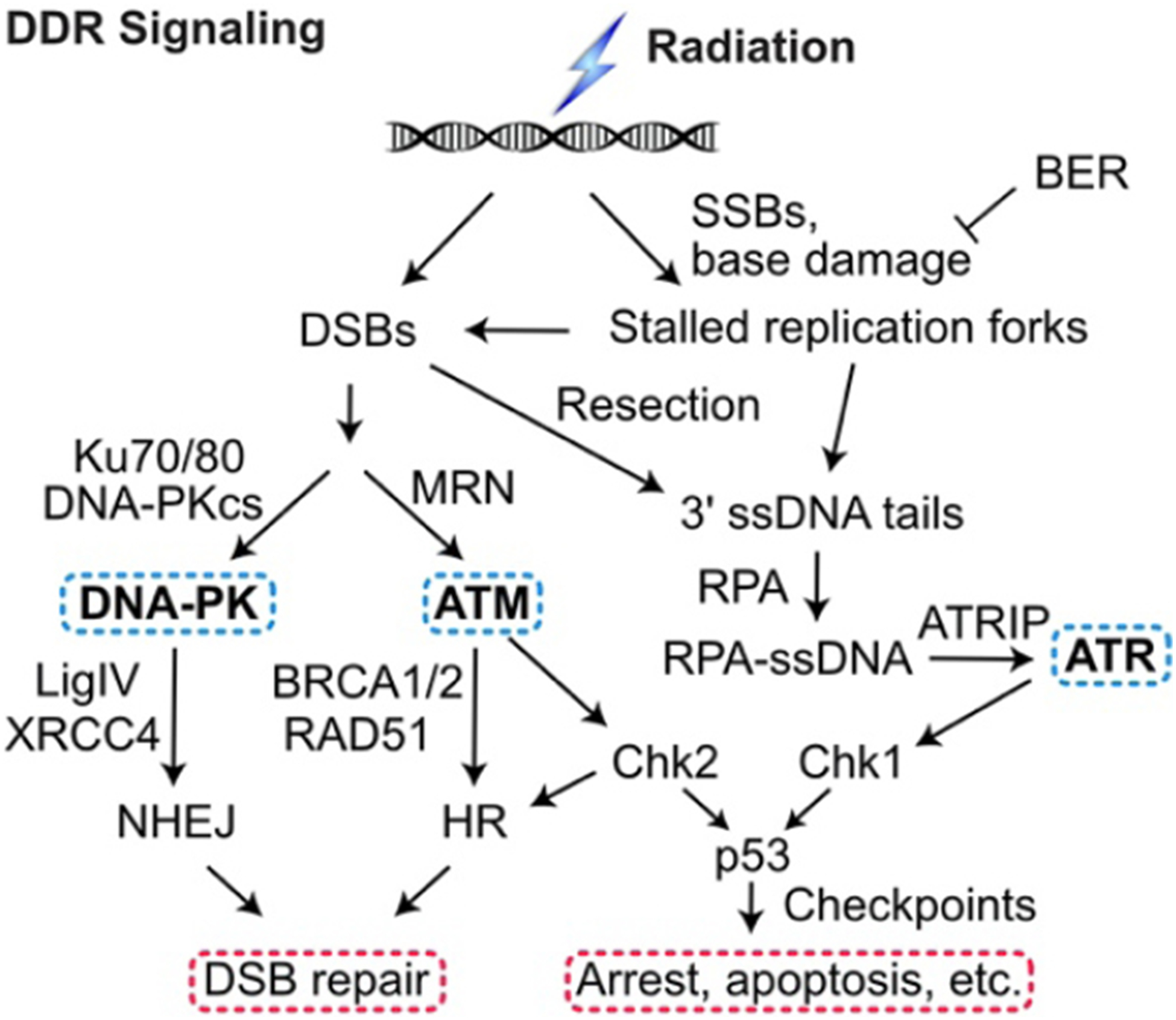
DDR signaling. Ionizing radiation and genotoxic chemotherapy create single- and double-strand DNA damage including DSBs that activate three PIKKs: DNA-PK, ATM, and ATR. Single-strand breaks and base damage, if not repaired by base excision repair (BER), block replication, which produces ssDNA when the replisome decouples from the MCM helicase or stalled forks are cleaved to produce DSBs, which, along with frank DSBs, are resected to 3’ single-stranded tails that are coated by RPA. This activates ATR to signal checkpoint responses through Chk1 and p53. Non-resected DSB ends are bound by the Ku70/Ku80 heterodimer, which recruits and activates DNA-PKcs in the DNA-PK holoenzyme, LigIV/XRCC4 ligates DNA ends to effect NHEJ. The competing HR pathway initiates with limited DSB end resection by MRE11/RAD50/NBS1 (MRN), more extensive resection by Exo1 and Dna2, and RAD51 binding to ssDNA (mediated by BRCA1, BRCA2, and other proteins) to yield the RAD51-ssDNA nucleoprotein filament that effects HR. DDR: DNA damage response; DSBs: DNA double-strand breaks

The DDR includes two checkpoint signaling pathways, one centered on ataxia telangiectasia mutated (ATM), a kinase that responds to DSBs and one centered on ataxia telangiectasia and Rad3 related (ATR) kinase that is triggered by single-stranded DNA (ssDNA) generated by 5’−3’ resection of DSB ends and by decoupling of the replication machinery from MCM helicase at stalled replication forks^[36–39]^. ATM and ATR, along with DNA-PKcs, are PI3 kinase-like kinases (PIKKs) that are “early responders” to DSBs and replication stress. PIKKs phosphorylate large networks of proteins^[40–42]^ including the downstream effector kinases Chk1 and Chk2 that phosphorylate p53 and other targets to arrest the cell cycle in response to damage, promote DNA repair, and promote programmed cell death pathways when damage exceeds a threshold^[43–46]^ [Figure 1]. The DDR thus presents two broad targets to manipulate for therapeutic gain: inhibiting DNA repair sensitizes cells to damage and inhibiting checkpoint signaling prevents cell cycle arrest in response to damage, increasing replication stress, fork collapse to DSBs, genome instability, and cell death^[20,47–50]^.

**Figure 2.**
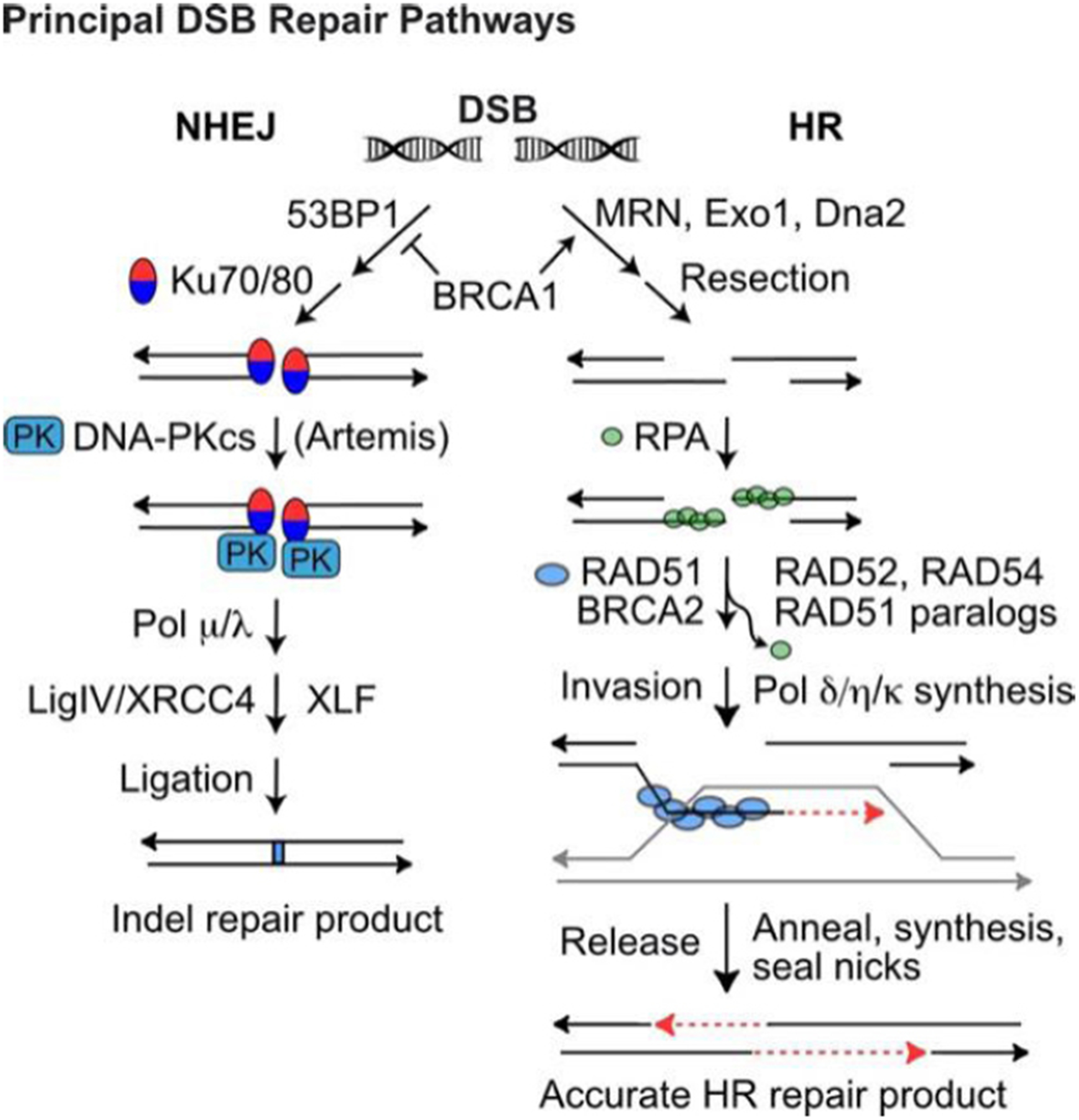
DSB repair by NHEJ and HR. Pathway choice is determined by end-resection, prevented by 53BP1, or promoted by BRCA1/MRN and Exo1/Dna2 exonucleases. (Left) NHEJ of unresected DSB ends initiates with Ku70/80 binding to ends and recruitment of DNA-PKcs to form the activated DNA-PK holoenzyme. Artemis is required to trim certain types of end-structures, and small gaps may be filled with polymerases *m* and *l* prior to LigIV/XRCC4/XLF-mediated ligation. NHEJ repair usually produces small indels (1–20-bp deletions, few-bp insertions). (Right) Resected 3’ single-strand ends are coated with RPA, which is then exchanged with RAD51, mediated by BRCA2, RAD52, RAD54, and RAD51 paralogs. The RAD51 nucleoprotein filament seeks and invades a homologous donor duplex (grey). RAD51 dissociates before repair synthesis; the newly synthesized strand (red dash) is released from the donor duplex and anneals with the complementary strand on the opposite side of the DSB. A second round of repair synthesis and nick sealing completes repair. DDR: DNA damage response; DSB: DNA double-strand break; NHEJ: non-homologous end-joining; HR: homologous recombination

DSBs are repaired by error-prone non-homologous end-joining (NHEJ) or by homologous recombination (HR) repair [Figure 2]^[51,52]^, templated from sister chromatids (restricted to S/G2 phases), homologous chromosomes, or short sequence repeats if the double-strand damage occurs within or nearby repeated sequences - not uncommon given the human genome comprises > 50% repetitive elements (Alu, MIRs, SINEs, LINEs, *etc*.)^[53]^. HR is generally accurate, but it does pose risks of genome rearrangements including large-scale loss of heterozygosity and translocations that can initiate tumorigenesis and drive tumor progression^[27,54,55]^. When the primary NHEJ or HR pathways fail, even more error-prone DSB repair pathways serve as back-up, including alternative (microhomology-mediated) NHEJ, single-strand annealing, and break-induced replication^[56–62]^.

## RADIOBIOLOGICAL PROPERTIES OF THERAPEUTIC IONIZING RADIATION

Three types of external beam radiation are used to treat cancer. X-rays and protons are low linear energy transfer (LET) radiation, although proton LET varies (see below). LET is a measure of ionization density, thus low LET X-rays (and protons for the most part) are sparsely ionizing. This means that most X-ray lesions, including DSBs, are widely dispersed. X-rays are massless photons that interact weakly with tissue, thus the highest X-ray doses are near the skin at the entrance point. To concentrate X-ray doses within tumors, beams are intensity modulated and delivered to patients from several angles, spreading low doses to a large volume of normal tissue^[3]^. Protons have a small mass and a single positive charge. Proton interactions with tissue slow and eventually stop these particles at a defined depth (within a tumor), termed the Bragg peak^[63]^. This feature provides a clear benefit as normal tissue beyond the tumor receives essentially no dose. Carbon ions with high mass and six positive charges are high LET radiation. Because of their mass, carbon ions also stop at depth and eliminate exit dose, similar to protons. However, the high mass and high charge of carbon ions produces dense ionization tracks, especially at the end of the track as particles slow and stop^[64,65]^.

X-rays, protons, and carbon ions induce the same number of DSBs per unit dose (~40 DSBs/Gy). Exposures to 1 Gy of X-rays or protons kills ~10%–20% of cells^[66–68]^. In contrast, the same dose of carbon ions kills 2–3-fold more cells, hence the relative biological effect (RBE) of carbon ions is ~2.5. Proton LET increases somewhat in the distal region of the Bragg peak, and RBE correspondingly increases to perhaps as high as 1.7^[65,69]^. The high RBE of carbon ions reflects the fact that these ions efficiently induce clustered DSBs, defined as two or more DSBs separated by < 200 bp^[64,68,70]^. Clustered DSBs are repaired inefficiently and are hence more cytotoxic than isolated DSBs. Low LET X-rays and protons induce occasional clustered DSBs - it is thought that these lesions primarily determine low LET radiation cytotoxicity, not the more prevalent isolated DSBs^[64,68,71–73]^. The greater cytotoxicity (RBE) of carbon ions reflects their greater efficiency at inducing clustered DSBs. NHEJ, the dominant DSB repair pathway, initiates with Ku70/Ku80 (Ku) binding to DNA ends and recruitment of DNA-PKcs [Figure 2]^[51]^. Ku appears to efficiently bind both large and small DNA fragments, generated by isolated and clustered DSBs, respectively. However, short fragments do not activate DNA-PKcs kinase^[74]^, which has critical roles in NHEJ, HR, DDR signaling, and checkpoint activation^[75]^. Thus, short DNA fragments appear to be refractory to repair by NHEJ, and this may account for both the greater cytotoxicity of clustered *vs*. isolated DSBs, and the shift from NHEJ toward HR in cells exposed to high LET radiation^[64,76–79]^. A greater dependence on HR was also observed with protons than X-rays^[80]^, perhaps reflecting the higher proton LET in the Bragg peak. However, a more recent study showed minimal differences when cells were treated with X-rays vs. protons, and inhibitors of NHEJ or HR^[81]^, suggesting additional factors determine repair pathway choices among cell types. That cells struggle to repair clustered DSBs may reflect their rarity in nature and the lack of selective pressure to evolve repair systems for this class of complex DNA lesion.

Low and high LET radiation are distinguished in two other ways. Low LET X-rays and protons induce ROS most efficiently in well-oxygenated tissue. At low oxygen levels, the cytotoxic effects of X-rays and protons is reduced ~3-fold, the so-called oxygen enhancement ratio (OER)^[82]^. Importantly, high LET carbon ions show far less reliance on oxygen (lower OER), owing to the greater ionization potential of these high mass/high charge ions^[82,83]^. Radiosensitivity varies during the cell cycle. Low LET X-rays and protons show highest cytotoxicity during G1 and M phases, and ~2-fold less cytotoxicity during S-phase, termed S-phase radioresistance^[84]^. Interestingly, high LET carbon ions show the opposite effect: ~2–3-fold S-phase radiosensitivity relative to G1 cells (Kato, unpublished results). This suggests one mechanism by which mixed high and low LET exposures might yield synergistic cell killing^[85–87]^.

The highly damaging effects of high LET radiation initially raised concerns about the safety of carbon ions in radiotherapy^[88]^, but serious side effects occur no more often than with X-rays or protons^[89–91]^. This safety profile probably reflects the fact that high LET ions behave similarly to low LET X-rays and protons while traveling through (normal) tissue at high speed, gaining their high LET properties only when slowing and stopping at the end of their tracks (in tumors)^[63,92]^. Thus, carbon ion LET and RBE are relatively low in the entrance region and increase dramatically in the Bragg peak, and the most damaging effects are confined to the tumor volume^[63,93,94]^.

### Cellular radiosensitivity and radioresistance

Many physical, biological, and environmental factors influence cell responses to ionizing radiation, including those that determine the level and types of damage to cell components; cell state (proliferating or quiescent, cell cycle phase); DDR signaling and DNA repair capacity; propensity for programmed cell death; cellular “memory” of past adaptive exposure; and tissue macro- and microenvironments. For example, RB status influences intrinsic radiosensitivity among individuals^[95,96]^, and such biomarkers can be exploited to personalize radiotherapy treatment planning^[97,98]^. The physical natures of ionizing radiation (photon *vs*. particle and large *vs*. small mass/charge) determine lesion spatial distributions, reparability, and cytotoxicity. Nonetheless, as noted by Willers, Xia, and colleagues^[99]^, “there is no absolute resistance to radiation”. If enough radiation can be delivered, all tumor cells will be eradicated regardless of environmental, genetic, or metabolic factors. The practical limitation, of course, is collateral damage to normal tissue. Hence, any strategy that increases radiation dose to tumors, decreases doses to normal tissues, increases tumor-specific cytotoxic effects of radiation, or protects normal tissue from unavoidable exposure can improve therapeutic gain and/or reduce side effects.

### Hypoxia

An important environmental factor that regulates DNA damage induction is oxygen level, which varies among tumor types, within different regions of a tumor, and between tumor and normal tissue. Normal tissue is well-oxygenated, but tumors are often hypoxic as they struggle to supply oxygen during their rapid growth. To a degree, tumors adapt to the hypoxic state, for example, by stabilizing HIF1α, which regulates oxygen metabolism and angiogenesis via vascular endothelial growth factor, among other effects^[100]^. Although certain solid tumors are frequently characterized as “hypoxic”, e.g., head and neck and pancreatic cancers, it is now clear that most solid tumors have hypoxic regions. The degree of hypoxia is regulated by passive oxygen diffusion, creating somewhat stable oxygen gradients across tumor masses, and by transient effects such as altered perfusion by tumor vasculature^[100]^. Given the importance of oxygen for ROS production during irradiation (OER), hypoxic regions within tumors are naturally radioresistant; this is a particularly vexing problem given that normal (well-oxygenated) tissue may suffer greater ROS damage than adjacent tumors, reducing therapeutic gain. Several strategies have been proposed to mitigate hypoxia-related radioresistance, including modulation of dose fractionation, inflammatory responses, and hypoxia itself^[101,102]^. For example, investigators have explored hyperbaric oxygen to radiosensitize tumors, and tourniquets to promote normal tissue radioresistance, but these approaches have fallen out of favor^[3]^. Another idea is to mimic oxygen with agents such as nitroimidazoles, which radiosensitize hypoxic tumors. Although these are effective, clinical use has been restricted because of associated neurotoxicity^[103,104]^.

## CELL PROLIFERATION RATES

Solid tumors comprise rapidly growing (“bulk”) tumor cells and small numbers of so-called cancer stem cells (CSCs). Much of tumor sensitivity to genotoxic chemo- and radiotherapeutics reflects the fact that rapidly dividing, bulk tumor cells are more sensitive to DNA damage than most (non-dividing) normal cells. CSCs, similar to normal stem cells, divide more slowly than bulk tumor cells, hence CSCs are naturally radioresistant. Because CSCs are tumor-initiating cells that support both local tumor growth and seed distant metastases, CSC radioresistance is a significant barrier to durable chemo- and radiotherapy treatment responses^[105–107]^. Similar to CSCs, some tumor cells may be quiescent; tumor dormancy is seen locally and at metastatic sites, it can be induced by therapy, and it confers radioresistance^[108]^. Changing fractions of bulk, CSC, and quiescent tumor cells may cause regional variations in tumor radioresistance, complicating radiotherapy treatment planning.

### Hyperthermia

The sensitizing effects of hyperthermia have long been investigated *in vitro* and in pre-clinical models, but it has not yet advanced to clinical practice^[109]^. Hyperthermia alters tissue perfusion to mitigate hypoxia, and it has been debated whether it directly induces DNA damage, but there is clear evidence that it triggers DDR signaling and suppresses DNA repair^[110,111]^. Ultrasound waves generate heat, and this technology is being explored to induce local hyperthermia for tumor radiosensitization^[112]^.

### Radioprotectors and radiosensitizers

Because most radiation damage is induced indirectly through ROS, intrinsic and extrinsic modulation of cellular re-dox status strongly affects radioresistance. Re-dox mechanisms have been investigated to sensitize tumors and/or protect normal tissue during radiotherapy, including modulating NAD^+^, glucose, and other re-dox metabolic pathways; use of antioxidants (e.g., vitamins C and E) and isoflavones; use of Mn-porphyrin compounds such as manganese-dependent superoxide dismutase (Mn-SOD) mimetics; modulating superoxide dismutase; and modifying patient exercise routines^[113–119]^. Metformin, which reduces hypoxia by reducing oxygen consumption, and melatonin, a natural hormone with antioxidant and anti-inflammatory effects, are also under investigation for radiosensitization or protection^[120–123]^. Radiation countermeasures are designed to protect individuals from adverse effects of accidental or intentional (i.e., dirty bomb) total-body irradiation; these strategies may be useful for normal tissue protection during radiotherapy^[124–126]^.

### Adaptive responses

Cells exposed to a low dose of radiation and then subsequently challenged by a high, cytotoxic dose show enhanced survival compared to cells that did not receive a “priming” dose. This effect, termed the adaptive response, typically refers to enhanced cell survival, but radioadaptive responses have been observed with other endpoints, including chromosome aberrations, mutation, micronuclei formation, sister chromatid exchange, delayed genome instability, and cellular transformation^[127–133]^. These radioadaptive responses are transient, usually subsiding within 24 h of the priming dose. Several regulatory proteins are known to positively or negatively influence cell survival adaptive responses to radiation, including Mn-SOD, NFkB, p53, and NOX4, several of which are mediated by the anti-apoptosis factor survivin^[134–139]^. Adaptive responses may be problematic, for example, if CT scans used to locate tumors induce tumor radioresistance^[134]^, but other radioadaptive effects, such as immunomodulatory responses, may prove beneficial^[140,141]^. These transient radioadaptive responses are distinct from two other types of tumor adaptative responses to therapy: adaptive (upregulated) mutagenesis, which accelerates tumor evolution, and modulation of tumor microenvironments, both of which can drive tumor resistance to radio- and chemotherapy^[142,143]^.

## TARGETING DSB REPAIR TO ENHANCE RADIOTHERAPY

DSB repair is a major determinant of cellular radioresistance, and key NHEJ and HR proteins are attractive tumor radiosensitization targets. Because DNA repair and DDR systems are tightly integrated, radiosensitization can be achieved by interfering with these networks in a multitude of ways. In addition, “omics” analyses hold promise for personalizing radiotherapy doses based on radiation response profiles^[144]^. For additional perspectives on these topics, readers are referred to these recent reviews^[31,35,97,145–149]^. Current experimental and therapeutic options that target DSB repair and DDR factors are listed in Table 1 and discussed in the following sections.

## TARGETING NHEJ

DNA-PKcs is activated when complexed with Ku-bound DNA ends at DSBs, leading to phosphorylation of itself and other targets including Ku, RPA, and H2AX. DNA-PKcs autophosphorylation at two clusters (ABCDE and PQR, including T2609 and T2056 residues) is critical for subsequent NHEJ steps^[51,75,184]^, and DNA-PKcs inhibitors are strong radiosensitizers. However, because NHEJ is active in all nucleated cells, and cells need to repair spontaneous DSBs, inhibiting NHEJ non-specifically may adversely affect normal tissues, especially those within the radiation field. In certain solid tumors, such as ovarian and liver cancers, DNA-PK activity is elevated and this correlates with poor prognoses^[185,186]^. In these cases, DNA-PKcs inhibition may improve therapeutic gain. Several small molecule DNA-PKcs inhibitors, and other targeted approaches, have shown promising results *in vitro* and in pre-clinical models to enhance radio- and/or chemotherapy, but few have advanced to human clinical trials, due at least in part to challenges associated with cross-inhibitory effects against PIKKs (ATM, ATR, and mTOR) or bioavailability.

NU7441 is a fairly specific DNA-PKcs inhibitor that showed promising results as a radiosensitizer against nasopharyngeal and liver cancer^[150,151]^, and low concentrations of NU7441 enhance radiosensitivity of lung cancer cells to both X-rays and carbon ions^[152]^. Targeting DNA-PKcs with NU7441 in combination with the PARP1 inhibitor rucaparib radiosensitized Ewing sarcoma cells^[181]^. The DNA-PKcs inhibitor VX-984 radiosensitizes glioblastoma cells *in vitro* and in orthotopic tumors^[153]^. Two recently developed small molecule DNA-PKcs inhibitors are NU5455 and AZD7648. NU5455 is a highly selective DNA-PKcs inhibitor that increases the efficacy of radiotherapy and genotoxic chemotherapy treatment of lung cancer xenografts^[154]^. AZD7648 is a highly selective and potent DNA-PKcs inhibitor that enhances radiotherapy of lung tumor xenografts alone and when combined with the PARP1 inhibitor olaparib; this drug is advancing to clinical trials^[182]^. Precise selectivity is not necessarily required: the DNA-PKcs inhibitors, LY3023414 and CC-115, cross-inhibit mTOR (another PIKK) and show promising pre-clinical results. LY3023414 has advanced to clinical trials^[155,156]^. In preclinical studies, selective radiosensitization of hypoxic tumors was achieved using the hypoxia-activated pro-drug BCCA621C to inhibit DNA-PKcs^[157]^.

Many tumors overexpress wild-type or mutant versions of the epidermal growth factor receptor (EGFR). The EGFR pathway feeds into the PI3K/AKT/mTOR pathway that drives cell cycle progression. Interestingly, EGFR pathway activation stimulates DSB repair, and this was traced, at least in part, to an interaction between AKT1 and DNA-PKcs^[187]^. In a parallel EGFR pathway, radioresistance of tumor cells that overexpress Rab5C, Ku70, and Ku80 was traced to Rab5C regulation of EGFR internalization and its translocation to the nucleus, where EGFR stimulates Ku70/Ku80 expression^[188]^. Cetuximab, a clinically useful monoclonal antibody that targets EGFR, inhibits DNA-PKcs^[158]^ and enhanced radiotherapy in early clinical trials to treat cutaneous squamous cell carcinoma^[159]^. EGFR nuclear translocation is stimulated by radiation mediated by Cavelolin-1 (CAV-1), and CAV-1 knockdown radiosensitizes triple-negative breast cancer, a tumor type for which there are no current targeted therapies and poor prognoses^[189]^.

Mutant forms of EGFR (D746–750, L858R, and the targeted-therapy resistant T790M mutant) confer radiosensitivity to hypoxic lung cancer cells, at least in part due to downregulation of RAD50, a member of the MRE11/RAD50/NBS1 complex that plays early end-processing and signaling roles in NHEJ and HR^[190]^. These results suggest that tumor EGFR status can be used to personalize radiotherapy treatment plans and augmentation with NHEJ inhibitors. The link between EGFR and DSB repair suggests strategies to modulate tumor radiosensitivity by inhibiting NHEJ indirectly with available drugs that target EGFR and AKT1/3 pathways^[148,160]^.

## TARGETING HR

A key step in HR is formation of RAD51 nucleoprotein filaments that seek and invade homologous duplex DNA repair template [Figure 2]. RAD51 sub-nuclear foci are observed ~1 h after irradiation and are often interpreted as evidence of “HR activity”. However, RAD51 nucleoprotein filament formation marks only the initial phase of HR; once the filament invades a donor duplex, RAD51 must dissociate to allow extension of the invading strand by repair-associated DNA polymerases^[191]^. Thus, RAD51 foci are markers of HR initiation, but persistent RAD51 foci may reflect failure to complete HR due to downstream HR defects^[192]^. Functional HR, therefore, is best assayed by directly detecting HR products. There are several types of HR assay systems, including plasmid transfection systems, integrated HR repeat substrates, and HR-mediated gene editing^[193,194]^. When assaying RAD51-dependent HR using linked (direct or inverted) repeats, it is important that the design detects RAD51-dependent gene conversion but not RAD51-independent single-strand annealing^[62]^. Plasmid transfection assays are convenient, but substrates may not be chromatinized before or during HR, and therefore may not accurately reflect the full constellation of HR functions in chromatin^[195]^. Similarly, gene editing involves transfection of a non-chromatinized, homologous donor DNA sequence. Plasmid and gene editing assays are useful in rapid HR screens that can be complemented by analysis of HR products in a chromosomal context.

**Figure 3.**
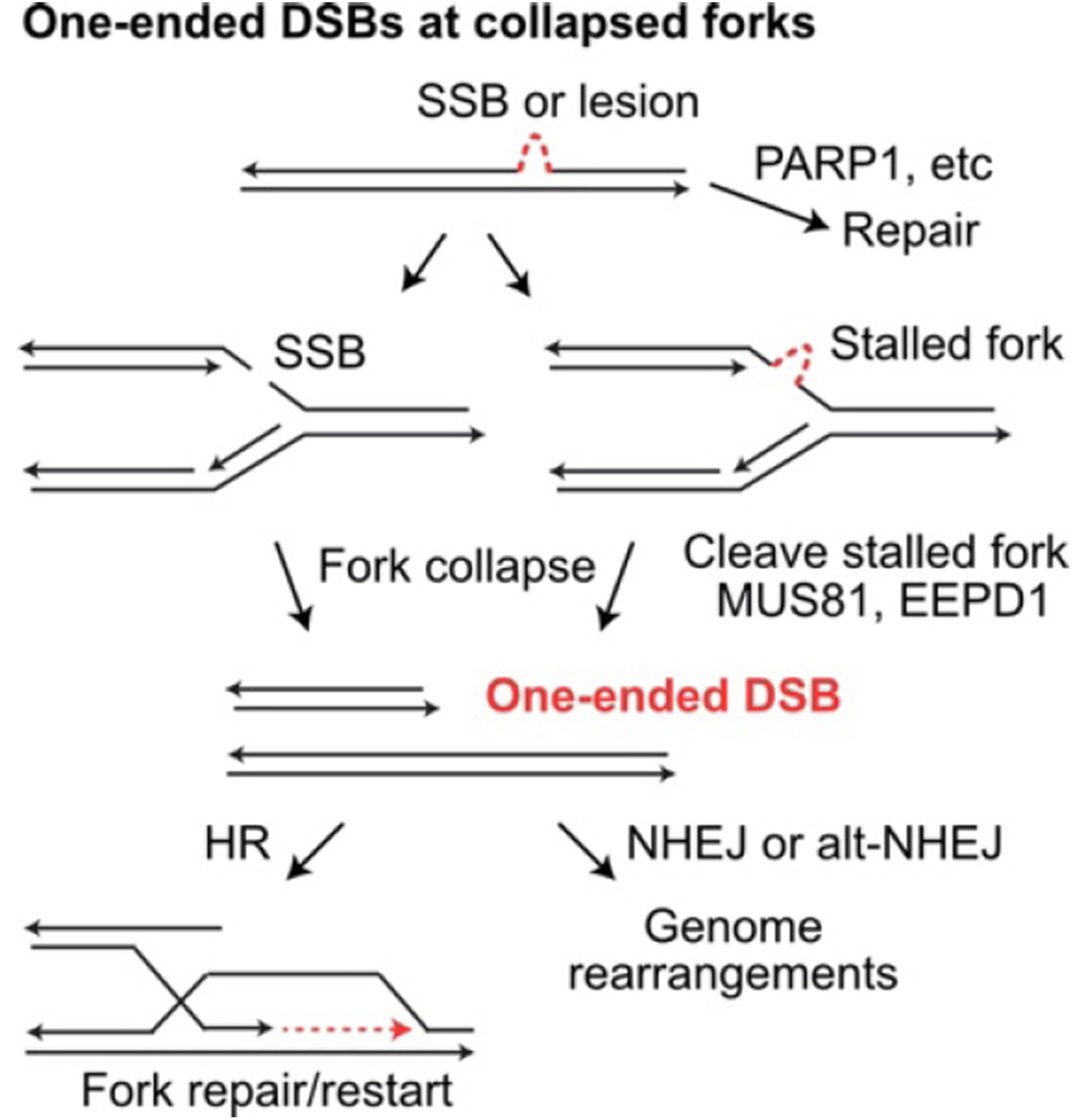
Repair and mis-repair of one-ended DSBs at collapsed replication forks. Single-strand breaks (SSB) and single-strand DNA lesions may be repaired prior to replication. If encountered by a replication fork, SSBs and single-strand lesions cause fork collapse and fork stalling, respectively. Stalled forks may collapse when cleaved by MUS81 or EEPD1. Collapsed forks create one-ended DSBs that can initiate fork restart by HR, or they may be joined to other DSB ends (at collapsed forks or frank DSBs) by NHEJ or alt-NHEJ, creating large-scale genome rearrangements. DSBs: DNA double-strand breaks; HR: homologous recombination; NHEJ: non-homologous end-joining

HR is important for repair of frank DSBs, but its other critical role is repairing single-ended DSBs that arise when replication stress causes fork collapse [Figure 3]^[196]^. A 1-Gy dose of ionizing radiation induces ~40 frank DSBs, but hundreds of single-strand lesions that can cause “secondary DSBs” due to fork collapse^[197,198]^. HR is critical for repair of these one-ended DSBs because mis-repair by NHEJ necessarily involves a distant DSB end (from a different broken replication fork or a frank DSB), causing large-scale genome rearrangements including deletions, translocations, and dicentric chromosomes that can trigger cell death or genome instability from persistent bridge-breakage-fusion cycles^[199]^. Thus, care must be taken when interfering with HR to enhance radiotherapy, as HR is critical for maintaining genome stability in normal tissues to prevent induction of secondary cancers.

Because RAD51 plays a central role in HR, it is an attractive target for radiosensitization. The Bishop and Connell labs developed a small molecule RAD51 inhibitor, RI-1, that blocks RAD51 binding to ssDNA^[200]^ and radiosensitizes glioma and glioblastoma cells^[161,162]^. New RAD51 inhibitors have been developed, including one that blocks D-loop formation (strand invasion) and HR but does not affect RAD51 binding to ssDNA or formation of radiation-induced RAD51 foci^[201,202]^. A recently developed antibody fragment linked to a cell-penetrating peptide blocks RAD51 DNA binding, sensitizes cells to radiation, and is synthetically lethal with PTEN defects in glioma and melanoma cells^[163–165]^. Another small molecule RAD51 inhibitor, CYT-0851, is currently in a clinical trial as monotherapy against several types of cancer^[166]^.

HR defects pre-dispose to cancer, including breast, ovarian, and other cancers with defects in BRCA1, BRCA2, PALB2, MRE11, and RAD51, as well as DDR factors that regulate HR, such as ATM^[203–206]^. HR proteins function as tumor suppressors by maintaining genome stability by promoting accurate DSB repair, stabilizing stressed replication forks, and repairing and restarting collapsed replication forks^[207]^. PARP1 inhibitors cause replication stress by inhibiting PARP1-dependent repair of single-strand damage and from PARP1-trapping on damaged DNA, accounting for the synthetic lethality of PARP1 inhibitors in HR-deficient tumor cells^[35,208]^. PARP1 inhibitors are widely used in clinical management of HR-defective breast and ovarian cancers^[209,210]^ and are being explored as adjuncts to radiotherapy^[168–170]^. To inhibit proteins such as BRCA2 for which there are no small molecule inhibitors, genetic approaches such as siRNA knockdown offer another means to transiently induce HR defects to enhance radiosensitivity^[167]^. HR defects, whether intrinsic to the tumor or induced by drugs or other means, may be particularly useful when paired with high LET carbon ions given the greater importance of HR in repair of clustered DSBs^[64,76–78,167]^. Just as HR defects sensitize cells to radiation and genotoxic chemotherapy, therapeutic resistance to these agents, and to PARP1 inhibitors, correlates with restoration or upregulation of HR^[211–215]^. Radiosensitization of tumors with HR inhibitors may thus be most effective against cancers that upregulate HR.

## TARGETING DDR SIGNALING FACTORS

The DDR is important for tumor suppression, and it also comprises important targets that mediate therapeutic resistance to radiation and chemotherapy^[216]^. ATM and ATR are key regulators of critical HR factors, including MRE11, NBS1, CtIP, p53, RPA, BRCA1, PALB2, H2AX, and RAD51^[37,192,217,218]^. ATM, ATR, and DNA-PKcs collaborate to regulate HR, NHEJ, and DNA damage checkpoint responses^[30]^. Targeting these PIKKs and other DDR factors, including Chk1, Chk2, and Wee1, are very active research topics^[35,97,146,148,149,219]^. Some DDR inhibitors show significant toxicity, hence delivery during protracted, fractionated radiotherapy raises safety concerns; these might be mitigated by using localized drug delivery. Nonetheless, several DDR inhibitors have advanced to clinical trials, including two phase 1 trials to augment radiotherapy with the ATR inhibitors VX-970 and AZD6738^[34,220]^. ATM inhibitors, including AZD1390 and AZD0156, have shown promise for radiosensitizing various solid tumors in preclinical studies, including glioblastoma, head and neck cancer, and lung cancer^[34,221–223]^. ATM and ATR inhibitors are also being tested for synthetic lethal effects with PARP1 inhibitors^[220–222]^; such combinations may also augment radiotherapy. The PI3K/AKT/mTOR pathway has well-defined roles in suppressing apoptosis and promoting cell proliferation, but it also interfaces with the DDR, promoting both HR and NHEJ^[171]^. PI3K/AKT/mTOR inhibitors sensitize tumor cells to PARP1 inhibitors^[224,225]^ and to radiotherapy^[172]^. HPV, the causative agent for most cervical cancers, modulates the DDR to confer therapeutic resistance, and DDR inhibitors are being explored to improve cervical cancer outcomes^[226]^. HPV is not alone: many viruses hijack different parts of the DDR to complete their life cycles^[227]^. ATM, ATR, and Chk1 signaling modulates PD-L1 expression in response to DSBs induced by radiation or chemotherapeutics^[141]^. In preclinical studies, inhibition of ATM during radiotherapy enhanced tumor immunogenicity and tumor sensitivity to PD-L1 immune checkpoint blockade^[183]^. These findings highlight the pleiotropic effects of PIKK signaling networks and suggest new opportunities for combination therapy to radiosensitize tumors and exploit anti-tumor activity of the immune system.

## SIMULTANEOUS TARGETING OF NHEJ AND HR WITH HSP90 INHIBITORS

Given the importance of DSB repair for cell survival, and the central roles of NHEJ and HR in DSB repair, simultaneously blocking these pathways can exquisitely sensitize tumors to radio- and chemotherapy. Hsp90 inhibitors have emerged as important tools for simultaneous downregulation of NHEJ and HR. Hsp90 is a protein chaperone that regulates stress responses and tumor growth proteins, and Hsp90 inhibitors are being used to treat cancer in monotherapy and to augment traditional therapies^[228–230]^. Although Hsp90 is not mutated in tumor cells, it has an altered conformation and higher ATPase activity than in normal cells. Hsp90 inhibitors exploit this difference to selectively affect tumor cells^[174,229,231,232]^. The radiosensitizing effects of Hsp90 inhibitors to low and high LET radiation have been studied for more than a decade^[174–179]^. The Hsp90 inhibitor 17-AAG, suppresses HR^[176]^, radiosensitizes tumor cells, and suppresses tumor growth after radiotherapy^[174]^. Interestingly, the greatest radiosensitization was observed with carbon ions^[174]^, another example of how HR inhibition potentiates radiosensitization with high LET radiation. Because protein chaperones affect many cellular processes, Hsp90 inhibitors can have pleiotropic effects, and early Hsp90 inhibitors caused serious side effects including ocular degeneration^[233,234]^. Second and third generation Hsp90 inhibitors (PU-H71 and TAS-116) proved to be safer alternatives. These drugs are tumor-specific radiosensitizers that suppress both NHEJ and HR by downregulating RAD51, RAD51 foci, and DNA-PKcs Ser2056/Thr2609 phosphorylation^[175,178]^. TAS-116 showed promising results in a phase 1 trial as monotherapy against advanced, heavily pre-treated gastrointestinal and lung cancers, with an acceptable safety profile (e.g., no greater than grade 1 ocular disorders and nausea) and anti-tumor activity^[180]^. It will be interesting to test TAS-116 as an adjunct to radiotherapy, and to carbon ion radiotherapy in particular.

## SUMMARY AND FUTURE PERSPECTIVES

DDR signaling, DNA repair, and DNA replication systems are tightly integrated, and they are key regulators of genome integrity, genome replication, and cell viability/cell proliferative capacity. This means that agents that target DDR and DNA repair factors can be highly effective against tumors, especially when exploiting a tumor-specific synthetic lethal weakness. Unfortunately, these systems are also critical in normal cells, and DDR and DNA repair inhibitors can cause unacceptable normal tissue damage, especially if delivered systemically, reducing patient quality of life, both short- and long-term, and potentially reducing lifespan due to organ failure, accelerated tumor progression, or secondary cancers. This delicate balance is exemplified by a recent study showing that ATM counters toxic NHEJ at collapsed replication forks - an important finding because it points to new synthetic lethal approaches to treat ATM-defective tumors^[235]^. However, it also raises the possibility of ATM inhibition enhancing NHEJ-mediated mis-repair of single-ended DSBs during (therapy-induced) replication stress. This would destabilize the genome and may accelerate progression of surviving tumor cells or induce secondary cancers.

Once radio-modulators are proven effective in pre-clinical studies, it is important to determine safe and effective ways to administer to patients. These will vary depending the type of radio- or chemotherapy being augmented, the types of agents administered, tumor location, and the organs at risk. Therapeutic efficacy can be increased, and side effects decreased, by employing multi-targeted approaches^[236]^. For example, the Li lab combined physical (radiation) targeting with two other targeting approaches. The first was an oncolytic adenovirus to deliver hTERT promoter-driven E1a gene for conditional replication in hTERT-positive (tumor) cells, and the second was a replication-defective adenovirus expressing shRNA to repress DNA-PKcs^[237]^. This downregulated NHEJ specifically in tumor cells within the (physically-targeted) radiation beam. Another tumor-specific targeting approach is illustrated by recent studies targeting triple-negative breast cancer. Here, CRISPR/Cas9 designed to knock out the Lcn2 oncogene was delivered to breast cancer cells using a tumor-tropic, ICAM1 antibody-linked nanomaterial^[238,239]^. These and other targeting strategies can be combined to enhance a wide variety of therapeutic interventions.

**Figure 4.**
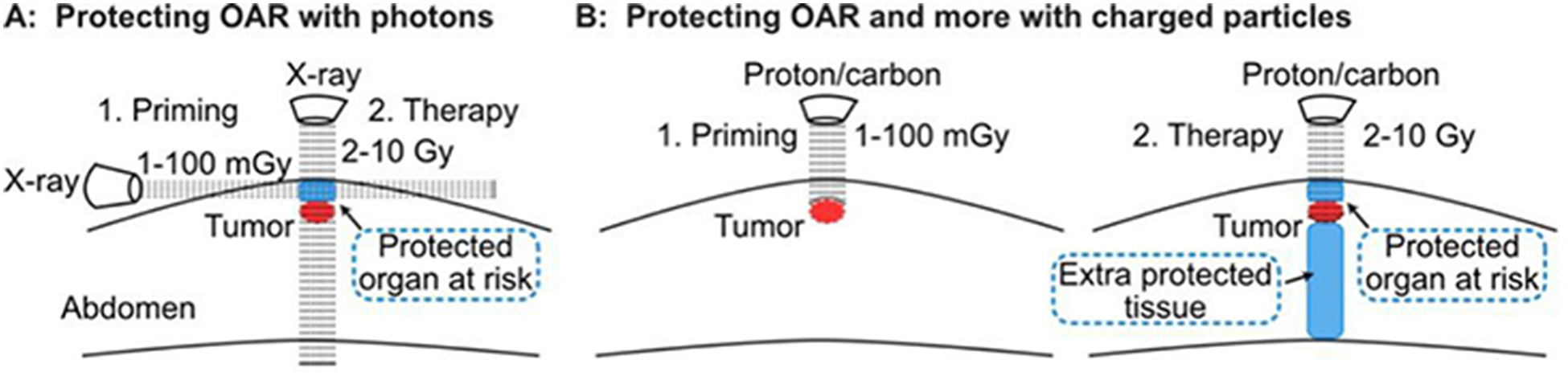
Proposed approach to protect normal tissue by stimulating radioadaptive responses. Horizontal X-ray beam delivers a priming dose to protect OAR (blue), but not the tumor (red) from subsequent, high doses delivered with a vertical beam(A); low priming dose of charged particles (left) protect OAR (blue) from subsequent high doses (right) (B). With charged particles, priming and therapeutic doses can be delivered along the same beamline since particles stop at predetermined depths. Charged particles also protect normal tissue distal to the tumor (larger blue section). OAR: organs at risk

The adaptive response raised concerns about improved tumor cell survival when tumors are “primed” with 5–10 mGy diagnostic CT scans to localize tumors before treatment with a 2–10 Gy “challenge” (therapeutic) dose^[134]^. It may be possible to invert this paradigm and exploit the adaptive response to protect normal tissue and increase therapeutic gain. This might be done, for example, by using a transverse photon (X-ray) beam to expose normal tissue above the tumor to low (mGy) doses. This could induce a transient adaptive response in at-risk normal tissue [specifically, organs at risk (OAR)], protecting this tissue from high dose radiotherapy delivered with a perpendicular beam [Figure 4A]. Such a strategy might be optimized with particle radiation, as priming doses can be delivered to just the normal tissue region that will be subsequently exposed to therapeutic doses in the entrance region, and particles also spare distal tissue [Figure 4B].

In conclusion, multi-targeted strategies that combine DNA repair and DDR-modulated tumor-specific radiosensitization, advanced photon and particle beam focusing, and radioprotection of normal tissues are a rational path to tumor cures with minimal side effects.

## Figures and Tables

**Table 1.** DSB repair and DDR targets to enhance radiotherapy

Pathway(s)	Target proteins	Inhibitors*	Ref.
NHEJ	DNA-PKcs	LY294002, LY3023414, NU7026, NU7441, NU5455, VX-984, CC-115, BCCA621C, IC86621, IC87102, IC87361, OK-1035, SU11752, KU-00600648	[150–157]
	EGFR	Cetuximab	[148,158–160]
HR	RAD51	RI-1, B02, CYT-0851, 3E10 (antibody), Fab-F2-iPTD (antibody fragment)	[161–166]
	BRCA2	BRCA2 siRNA	[167]
	PARP1	AZD2281, olaparib, veliparib, rucaparib, PJ34, E7016, AG14361, GPI-15427, 4-amino-1,8-naphthalimide	[168–170]
DDR	ATM, ATR, Chk1/2, Wee1	KU-55933, KU-60019, KU-59403, CP466772, AZD0156, VX-970, VE-821, AZD-6738, (−)-Schisandrin B, NVP-BEZ235, ETP-46464, AZ-20, AZD-7762, PF-00477736, XL-844, SCH-900776, prexasertib (LY2606368), LY2880070, SRA737, GDC-0575, AZD-1775, CJM061	[34]
	PI3K/AKT/mTOR	CC-115, BEZ235, PI103, BKM120, rapamycin, NVP-BEZ235	[156,171–173]
Multiple	Hsp90 (DNA-PKcs + RAD51)	17AAG, PU-871, TAS-116	[174–180]
	DNA-PKcs + PARP1	Rucaparib + NU7441, AZD7648 + olaparib,	[181,182]
	ATM/ATR/Chkl + PD-L1	shRNA-ATM + PD-L1 antibody	[183]

* Inhibitors differ in potency, specificity, and pharmacological properties that determine whether they are restricted to research purposes or appropriate to advance to pre-clinical and clinical investigations. DDR: DNA damage response; DSB: DNA double-strand break; NHEJ: non-homologous end-joining; HR: homologous recombination; EGFR: epidermal growth factor receptor
